# A Novel Robust Screening Assay Identifies *Pseudomonas* Strains as Reliable Antagonists of the Root-Knot Nematode *Meloidogyne incognita*

**DOI:** 10.3390/microorganisms11082011

**Published:** 2023-08-04

**Authors:** Tobias Stucky, Miro Hochstrasser, Silvan Meyer, Tina Segessemann, Andrea Caroline Ruthes, Christian H. Ahrens, Cosima Pelludat, Paul Dahlin

**Affiliations:** 1 Entomology and Nematology, Plant Protection, Agroscope, Müller-Thurgau-Strasse 29, 8820 Wädenswil, Switzerland; 2 Method Development and Analytics, Agroscope, Reckenholzstrasse 190, 8046 Zürich, Switzerland; 3 Mycology, Plant Protection, Agroscope, Route de Duillier 60, 1260 Nyon, Switzerland; 4 Swiss Institute of Bioinformatics—SIB, Reckenholzstrasse 190, 8046 Zurich, Switzerland; 5 Virology, Bacteriology and Phytoplasmology, Plant Protection, Agroscope, 1260 Nyon, Switzerland

**Keywords:** *Pseudomonas*, *Meloidogyne incognita*, root-knot nematode control, antagonist, biological nematicide, complete genome, phylogenetic analysis

## Abstract

Forty-four bacterial strains isolated from greenhouse soil and beetroots were tested for their antagonistic activity against the plant-parasitic root-knot nematode (RKN) *Meloidogyne incognita*, which causes significant yield losses in a number of important crops worldwide. Through a novel combination of in vitro and on planta screening assays, *Pseudomonas* spp. 105 and 108 were identified as the most promising bacterial isolates. Both strains were evaluated for their potential to control different RKN population densities and as root protectants against nematode infestation. Regardless of the application method, both strains significantly reduced root galling caused by *M. incognita*. These two strains were subjected to whole genome sequencing and de novo genome assembly as a basis for phylogenetic and future functional characterization. Phylogenetic analysis revealed that both *Pseudomonas* strains cluster within the *Pseudomonas fluorescens* clade among previously characterized RKN antagonists and *Pseudomonas*-based biocontrol agents of plant diseases.

## 1. Introduction

The plant-parasitic nematode *Meloidogyne incognita* is one of the most common root-knot nematode (RKN) species worldwide [1,2]. This group of obligate, sedentary plant parasites has a wide host range and can cause significant damage to several important agricultural and greenhouse crops, monocots, and dicots [3,4]. *M. incognita* infections can cause reduced plant growth, stunting, leaf discoloration, and even complete crop loss. The current estimate of the global yield losses caused by plant-parasitic nematodes places the annual losses in the range of billions of dollars [5], higher than those estimated for insects [6,7,8] and accounting for 14% of the total global crop losses [9].

RKN can reproduce both sexually (amphimixis) and asexually (mitotic and meiotic parthenogenesis). During their life cycle, second-stage juveniles (J2) enter the root tip near the elongation zone and migrate intercellularly to establish a permanent feeding site by inducing the formation of giant cells in the vascular cylinder [10]. While depleting plant nutrients and causing root galling, the juveniles molt and develop a swollen, pear-shaped appearance. Mature female nematodes lay their eggs in a gelatinous matrix outside the root system, allowing the J2 to hatch freely in the soil and find new root tips for propagation. Apart from the adult males, only the infective J2 are mobile and can move in the soil after hatching (pre-parasitic stage). During this exophytic phase, RKN spend a long time in the soil before infecting a host root [11].

The exophytic phase is important for both the synthetic chemical and biological control of RKN. For example, soil fumigation has often been used as a rapid, “reliable” means of controlling RKN in soil [12]. Several nematicides are contact nematicides and are applied outside the vegetative period as they can negatively affect seed germination and plant growth. Due to human health and environmental safety concerns, most chemical nematicides are no longer authorized or are strictly regulated in Switzerland and most other countries worldwide [12,13].

In biological control, J2 nematodes in the soil can be antagonized by predators, endoparasites, egg parasites, hyperparasites, and microorganisms in general [14,15]. Such microorganisms have been described to confer nematode suppressiveness to soils [16] and may consist of one or a complex of antagonistic species [17]. Fungal species (e.g., *Dactylellina* spp., *Drechslerella* spp., or *Arthrobotrys* spp.) [18] and bacterial species (e.g., *Arthrobacter* spp., *Burkholderia* spp., *Lysobacter* spp., *Pasteuria* spp., *Pseudomonas* spp., *Rhizobium* spp., and *Streptomyces* spp.) [19] have also been described to control plant-parasitic nematodes. The genus *Pasteuria*, which is known to attach to the cuticles of nematodes using an endospore, has been intensively studied as a potential bacterial antagonist of nematodes [20]. *Pseudomonas* spp. are also widely exploited as antagonistic microorganisms. Some *Pseudomonas* species are able to utilize plant root exudates for nutritional purposes and produce protective metabolites against pests and pathogens, as well as antimicrobial compounds known to control plant-parasitic nematodes [21,22]. In vitro and on planta studies have shown that *Pseudomonas* cell suspensions or supernatants can be successfully used to control RKN such as *M. incognita* and that their RKN control can be transferred to greenhouse conditions [23,24,25].

*Pseudomonas* spp. are commonly found in natural environments, colonize soil and plant root systems, and are easily maintained and cultured in the laboratory [26,27].

Screening for additional/alternative RKN antagonistic bacteria in the soil rhizosphere may be a promising method for the integrated management of plant-parasitic nematodes. Evaluating the potential of these isolates as RKN antagonists in a screening system that mimics the natural infection process of nematodes could improve the search for potential antagonists and allow the identification of particularly promising isolates.

Therefore, the objectives of this research were to (1) uncover a rapid and reliable method for the screening of nematicidal bacteria, in this proof-of-principle study for potential antagonists of *M. incognita*; (2) evaluate the selected strains for their nematicidal activity in the greenhouse; and (3) compare the phylogenetic relationships of promising antagonist strains with previously characterized *Meloidogyne* spp. antagonists to better select and understand potential candidates for RKN control in future trials.

## 2. Materials and Methods

### 2.1. Rearing and Collection of Meloidogyne incognita Second-Stage Juveniles

*M. incognita* was propagated on tomato (*Solanum lycopersicum*) cv. Oskar under greenhouse conditions (25 °C/19 °C, 60% humidity, 15/9 h light/dark cycle). Second-stage juveniles (J2) were extracted from heavily galled root systems using a mist chamber (23 °C) [28]. Hatched J2 were collected daily and stored at 6 °C for 3–7 days until used in experiments. Total DNA was extracted periodically for barcoding analysis according to Kiewnick et al. [29], to ensure *M. incognita* identity.

### 2.2. Bacterial Isolation and Culture Preparation

Beetroot (*Beta vulgaris* subsp. *vulgaris*, variety “Pablo”, grown in the greenhouse at Agroscope-Wädenswil (CH)) was harvested, the soil was removed, and small parts of the roots were resuspended in phosphate buffer (15 mM K_2_HPO_4_ and 9 mM KH_2_PO_4_, pH 7). After 10-fold serial dilution (up to 10^−3^), 100 µL of the respective dilution steps were plated on tryptic soy broth (TSB, Oxoid/Thermo Fisher Scientific, Waltham, MA, USA) agar plates (15 g/L Agar-Agar, Kobe I (Carl Roth, Karlsruhe, Germany)). Plates were incubated at 26 °C for 24 h; single colonies were picked and subcultured to ensure pure cultures. Bacterial isolates were identified at the genus level with MALDI-TOF by smearing bacterial cell material from a colony on agar plate onto a MALDI targe for the identification of specific biomarker peptides [30] and 16 S rRNA gene sequencing. Forty-four bacterial isolates (Supplementary Table S1) were then tested for their antagonistic potential against *M. incognita*.

### 2.3. Antagonistic Screening against Meloidogyne incognita

The bacterial isolates were freshly prepared on TSB (Oxoid/Thermo Fisher Scientific, Waltham, MA, USA) agar plates (15 g/L Agar-Agar, Kobe I (Carl Roth)). Each bacterial strain was cultured overnight at 26 °C in a 250 mL Erlenmeyer flask containing 100 mL TSB medium. Liquid cultures were centrifuged at 6000 rcf for 10 min at room temperature, supernatant decanted, and bacterial pellet resuspended in autoclaved tap water to a final optical density (OD_600nm_) of 0.5.

Nematode:bacteria mixtures were prepared by pipetting 6 mL of a water suspension containing 250 *M. incognita* J2 into a 25 mL plastic cup and adding 4 mL of the freshly prepared bacterial suspension to a final volume of 10 mL. The nematode:bacteria suspensions were incubated for three days at 20 °C in the dark. Thereafter, 20 mL of steamed soil:silver sand mixture (1:3, *v*/*v*) was added to the cups containing the nematode:bacteria suspensions. A three-day-old germinated cucumber seedling (*Cucumis sativus* cv. Sprinter F1) was then planted in each cup and grown in a climate chamber at 24 °C, 60% humidity, and a 16/8 h light/dark cycle and watered as needed (n = 3). After three weeks, cucumber roots were washed free of soil, and root gall formation caused by viable *M. incognita* J2 was determined according to Zeck [31]. After the initial screening, the same experimental setup was repeated for the pre-selected bacterial strains with nematicidal activity.

### 2.4. In Vitro Screening of Selected Bacterial Strains against Meloidogyne incognita Second-Stage Juveniles

The ability of the selected bacterial strains and the negative control *Pseudomonas orientalis* F9 [32] to inhibit *M. incognita* J2 was tested in vitro using 24-well plates (n = 3). Bacteria were processed as described above but adjusted to a final OD_600_ of 1.0. Each of the 24 wells was filled with 1 mL of a nematode suspension containing approximately 150 J2 and 1 mL of the OD_600_ = 1.0 bacterial suspension (OD_600_ of 0.5).

For the exposure of the J2 to the bacterial supernatant, the supernatant was filtered through a 0.2 μm syringe filter and 1 mL was added to a suspension containing 150 J2. The well plates were kept at 20 °C in the dark. For each well, the motility of the first 100 J2 individuals was scored under a light microscope as normal motility, affected motility, or immotile (elongated shape) [33].

### 2.5. Testing of Pre-Selected Antagonistic Pseudomonas Strains under Soil Conditions

The nematicidal activity of the bacterial isolates showing promising effects in vitro and on planta, as well as the control *P. orientalis* F9, was further tested under soil conditions in a greenhouse using cucumber plants as indicator plants. Pots (∅ = 14 cm) filled with 750 mL of steamed soil:silver sand mixture (1:3, *v*/*v*) were inoculated with ca. 4000 J2. Three days after nematode inoculation, 25 mL bacterial suspensions adjusted to an OD_600_ of 0.5 were prepared as described above and added to the nematode-infested soil, except for the bacteria-free controls (n = 8). The pots were then covered with plastic foil and kept moist. Seven days after the application of the bacteria, 3-week-old cucumber plants were planted in the soil. The treatments were arranged in a randomized block design in the greenhouse and kept at 25 °C/19 °C, 60% humidity, and a 15/9 h light/dark cycle. Plants were watered according to their needs. After 4 weeks, the plants were uprooted and washed with tap water to remove soil for root gall rating [31].

The experimental setup was repeated for the selected bacterial strains (n = 7), now using 4-week-old tomato cv. Moneymaker plantlets as indicator plants and testing the bacterial suspensions at OD_600_ = 0.5 and 1.5.

### 2.6. Evaluation of the Biocontrol Efficacy of Top Pseudomonas Isolates against Meloidogyne incognita by Application to Mineral Wool Plant Starting Plugs

Grodan SBS mineral wool plugs (SBS 36/77, Grodan, Roermond, The Netherlands) were used to grow cucumber seedlings. After 14 days of growth, the plugs were soaked overnight with 10 mL of selected bacterial suspensions (OD_600_ of 0.5). Bacteria-soaked cucumber plugs were then planted in 14 cm ∅ pots. Three days earlier, the soil:silver sand mixture of these pots had been inoculated with approximately 4000 *M. incognita* J2. Each treatment (selected *Pseudomonas* strains and control, *P. orientalis* F9) was replicated seven times (n = 7). Plants were arranged in a randomized block design in the greenhouse and grown at 25 °C/19 °C, 60% humidity, and a 15/9 h light/dark cycle. After four weeks, plants were uprooted and washed with tap water to remove substrates for root gall rating [31].

### 2.7. Evaluation of the Biocontrol Efficacy of Selected Pseudomonas Strains against Different Population Densities of Meloidogyne incognita

The top *Pseudomonas* strains were tested at low (2000 J2) and high (8000 J2) *M. incognita* soil concentrations (n = 8 for each treatment and nematode concentration, including 8 untreated control plants). Pots (∅ = 14 cm) were prepared with steamed soil:silver sand mixture (1:3 *v*/*v*) inoculated with J2. Bacterial suspensions were prepared as described above, but resuspended to a final OD_600_ of 1.5. Tomato cv. Moneymaker seedlings grown in transplanting trays for 4 weeks were planted in infected pots and arranged in a randomized block design. Tomato growth was measured weekly. Forty days after planting, root galling was evaluated according to Zeck [31].

### 2.8. Genomic DNA Extraction, Sequencing, De Novo Assembly, and Annotation

Total DNA was isolated from bacterial cells grown in TSB overnight according to [34]. The quality and quantity of the extracted DNA was assessed on a 0.8% (*w*/*v*) agarose gel, followed by the Qubit dsDNA GR assay (Life Technologies, Thermo Fisher Scientific, Waltham, MA, USA).

Both Illumina short-read and Oxford Nanopore Technologies long-read sequencing technologies were used. Illumina paired-end reads were trimmed with Trimmomatic v0.39 [35] (parameters: -phred 33 leading:3 trailing:3 sildingwindow:4:15 minlen:36), using FastQC v0.11.9 [36] to check the read quality before and after trimming. Oxford Nanopore raw sequence reads were filtered according to their quality and length using Filtlong v0.2.0 (https://github.com/rrwick/Filtlong, accessed on 29 July 2019). The filtered reads were de novo assembled using Flye v2.9.1-b1780 [37] with default parameters; the *dnaA* gene was chosen as the start position of the assembly. Assemblies were first polished with long reads using Medaka (https://github.com/nanoporetech/medaka, accessed on 7 October 2021) and then with trimmed Illumina reads and Freebayes v.1.3.2 [38] (minimum alternate fraction, 0.5; minimum alternate count, 5). Variants were manually inspected in Integrated Genome Viewer (IGV) [39] and subsequently corrected using BCFtools v.1.10.2 [40] to remove any potentially remaining sequencing errors. PlasmidSpades v3.13.1 [41] was run to assemble the Illumina short reads to detect short plasmids that may have been missed in the long-read-based assemblies. To verify the circularity and completeness of the de novo assemblies, long and short reads were mapped to each assembly using Minimap2 [42] and BWA-MEM v0.7.17 [43], respectively. Alignments were manually inspected using IGV. The mapping quality of reads was assessed using Qualimap v.2.2.1 [44]. The completeness of the final assembly was further assessed using BUSCO v5.0.0 [45]. As a final QC step, an in-house prototype for the detection of repeats was run to identify large repeats, some of which could indicate misassembled regions [46].

Finally, the finished genomes were annotated using the NCBI’s Prokaryotic Genome Annotation Pipeline (PGAP) [47].

### 2.9. Phylogenetic Analysis

A phylogenetic tree based on 70 *Pseudomonas* strains, including the two de novo assembled *Pseudomonas* strains 105 and 108, was calculated based on 16 core genes (*rpsJ*, *glnK*, *rplK*, *infA*, *rpsE*, *rplV*, *rplP*, *rpsG*, *rpsC*, *rpsR*, *rpsL*, *mreB*, *ihfA*, *rpsK*, *rplN*, *rpsU*) identified by Page et al. [48]. Multi-sequence alignment of all genes was performed with the ClustalW algorithm [49] using MEGA-CC [50], and a phylogenetic tree was inferred using the Maximum Likelihood method with MEGA-CC and 100 bootstrapping iterations. The 68 strains were selected based on a literature search of available *Pseudomonas* genomes with putative activity against nematodes, a previous study of *Pseudomonas* isolates with biocontrol potential that placed them in the context of known *Pseudomonas* subclades [51], and the five closest genomes of the two isolates reported here (based on sequence identity as estimated by Mash [52] of all publicly available strains on NCBI). All sequences were downloaded from the NCBI database.

### 2.10. Data Analysis

Statistical analyses were performed using the SPSS software, data were tested for homogeneity of variances (Levene’s test), and treatments were distinguished by one-way ANOVA with Tukey’s Honestly Significant Difference (HSD) post-hoc test (*p* ≤ 0.05). Results of the in vitro assay using cells and supernatant were expressed as percentages. Significant differences were calculated by comparing data from J2 with normal motility.

## 3. Results and Discussion

### 3.1. Identification of Bacteria with Antagonistic Activity against Meloidogyne incognita

#### Screening of Bacterial Strains with Potential Antagonistic Activity against Root-Knot Nematodes

**Figure 1:**
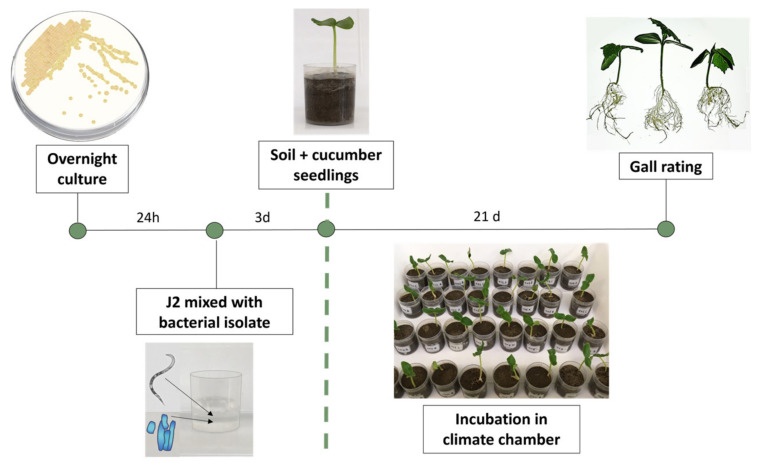
Schematic representation of the bacterial screening system against *M. incognita* second-stage juveniles (J2) using cucumber seedlings. Suspensions of the potential bacterial antagonists were prepared and mixed in a cup with 250 J2 in an aqueous solution. Nematodes and potential antagonists were incubated for 3 days before soil was added to the cup and a pre-germinated cucumber seedling was planted. After 21 days, cucumber roots were washed free of substrate and root galls were scored according to Zeck’s [31] 0–10 scale, where 0 indicates no galls and 10 indicates dead roots.

The antagonistic potential of 44 bacterial strains (Supplementary Table S1) against *M. incognita* was tested with a newly developed method using a combined “in vitro” on-plant cucumber assay (Figure 1). The screening method was designed to ensure that the J2 were exposed to the bacteria in an aqueous solution and also had the opportunity to infect a host plant. Therefore, in this method, an inhibitory effect of the tested bacteria on the J2 nematodes results in a reduction in or even the absence of root gall formation in the indicator plants. Thus, the effect of the bacteria on J2 plant infectivity can be assessed even if the J2 are visually unaffected. This is an advantage over studies with RKN antagonists, which rely solely on a visual assessment of the viability of the nematodes [53,54,55].

Preliminary characterization revealed that most of the bacterial isolates tested belonged to the genus *Pseudomonas* (Supplementary Table S1). *P. orientalis* F9 was included in the study to assess its activity against nematodes, as this strain is known to have antagonistic potential against the fire blight pathogen *Erwinia amylovora* and also against the oomycete *Pythium ultimum* [32].

**Figure 2:**
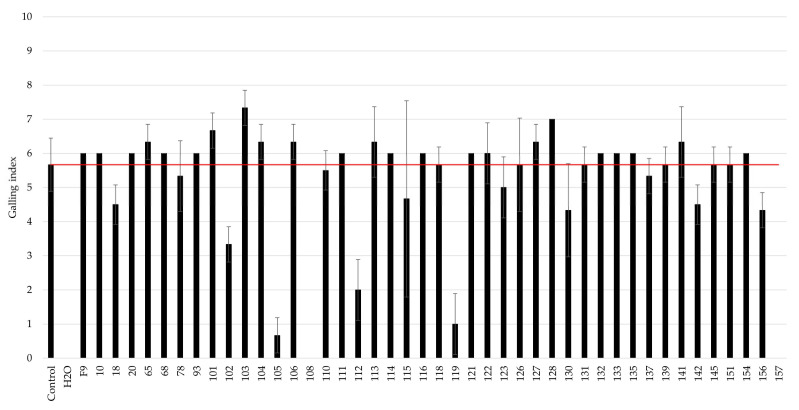
Screening of bacterial strains against *M. incognita* using an in vitro + cucumber bioassay. The galling index was scored according to Zeck’s [31] 0–10 scale, where 0 indicates no galls and 10 indicates dead roots, caused by *M. incognita*. The red line indicates root galling caused by *M. incognita*-untreated control, J2-infected soil without addition of bacterial strains. H_2_0 represents the negative control where no nematodes or bacteria were added to the cucumber seedlings. Error bars represent the standard deviations of the three replicates (n = 3) per treatment.

The antagonistic activity of the selected bacteria was determined by the root gall index recorded on cucumber roots caused by *M. incognita* J2 exposed to the selected bacterial strains and compared to an infection control, in which no bacterial antagonists were added to the J2-infected soil (Figure 2). The most promising antagonists of *M. incognita*, i.e., strains 102, 105, 108, 112, 119, and 157, caused a twofold or greater reduction in the galling index.

**Figure 3:**
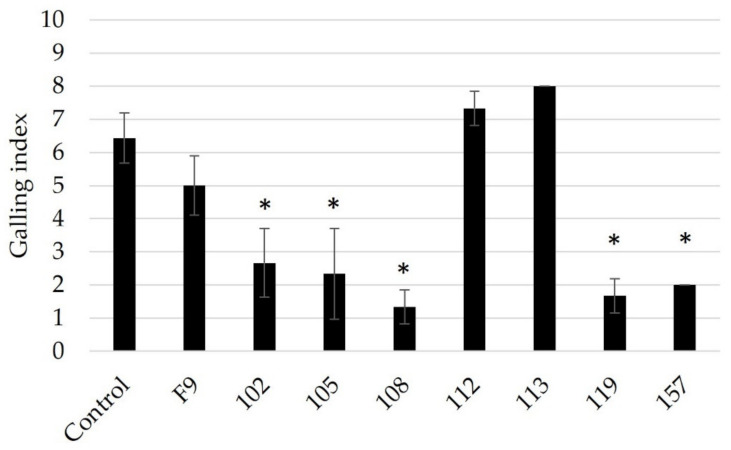
Testing of pre-selected *Pseudomonas* strains 102, 105, 108, 112, 119, and 157 against *M. incognita* using an in vitro + cucumber bioassay (n = 3). Infection control, only nematodes. Negative control for bacterial antagonism, *P. orientalis* F9 and isolate 113. Root gall index was scored according to Zeck’s [31] 0–10 scale, where 0 indicates no galls and 10 indicates dead roots. Error bars represent standard deviations of three replicates (n = 3) per treatment. Significant differences are indicated by an asterisk, calculated by a one-way ANOVA with post-hoc Tukey HSD test.

*Pseudomonas* strains (102, 105, 108, 112, 119, and 157) were retested for their ability to antagonize RKN (Figure 3). Based on the results of the initial screening, *P. orientalis* F9 and isolate 113 were added as negative controls for bacterial antagonism since neither strain showed inhibitory effects on RKN-induced galling (Figure 2).

With the exception of strain 112, strains 102, 105, 108, 119, and 157 repeatedly showed RKN antagonistic activity, while *P. orientalis* F9 and isolate 113 again had no significant effect on J2 (Figure 3). The results confirmed the reproducibility of the data obtained in the cucumber assay.

**Figure 4:**
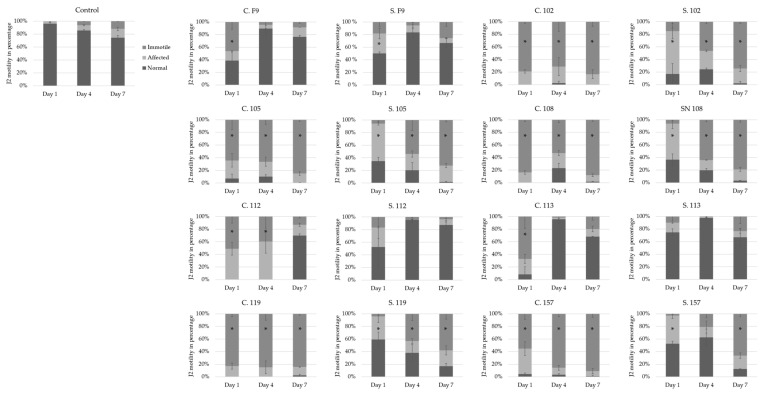
In vitro effect of different *Pseudomonas* strains applied as cells (C) or supernatant (S) on the motility of *M. incognita* second-stage juveniles (J2) after 1, 4, and 7 days. J2 motility was recorded according to normal motility, affected, or immotile nematodes [33]. Error bars represent standard deviations of replicates. * Significantly affected or immotile nematodes in percent (%) relative to the control, calculated using a one-way ANOVA with post-hoc Tukey HSD test (n = 3).

Bacterial cells and sterile filtered supernatants of *Pseudomonas* strains 102, 105, 108, 112, 119, and 157 were then tested against J2 in a pure in vitro assay to compare the antagonistic effects that occurred with the results obtained from the cucumber experiments. The 7-day in vitro assay showed that, for all strains, bacterial cells added to a J2 suspension significantly inhibited the nematodes after 1 day (Figure 4). However, 4 days (strains F9 and 113) or 7 days (strains F9, 112, and 113) after the application of the cell suspension, the J2 recovered and showed no significant differences compared to the water control. These results were even more pronounced when the supernatant of these strains was tested (Figure 4). Accordingly, the results support the notion that visual screening at a too early time point may give a misleading indication of RKN control in soil, as was observed with *P. orientalis* F9 and isolates 112 and 113. These negative controls in the cucumber assay significantly inhibited *M. incognita* J2 after one day when applied either as a cell suspension or its corresponding supernatant (Figure 4). In the literature, similar RKN in vitro assays using *Pseudomonas* spp. are typically evaluated after 12, 24, 48, and/or 72 h [56,57], and therefore some important information may be overlooked compared to the newly developed screening assay outlined in Figure 1.

### 3.2. Testing of Promising Meloidogyne incognita Bacterial Antagonists under Greenhouse Conditions

*Pseudomonas* strains 102, 105, 108, 119, and 157 were evaluated for their potential to control RKN under soil conditions. *P. orientalis* F9 was selected as a negative control for the soil experiment. The bacterial cultures were applied to *M. incognita*-infested soil and cucumbers were planted seven days after application.

**Figure 5:**
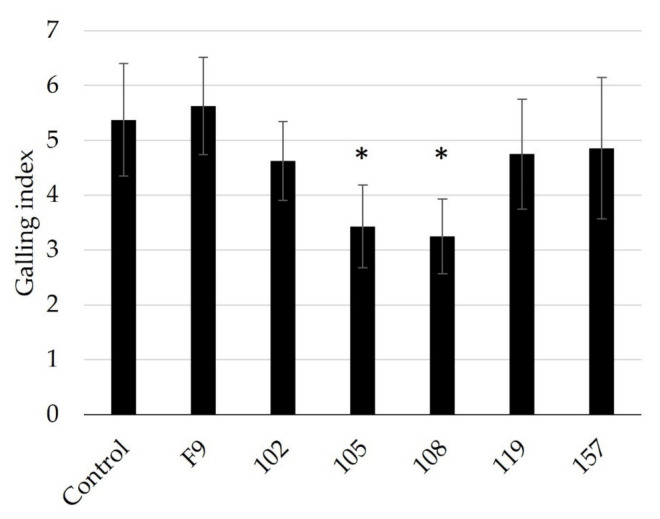
Testing of selected *Pseudomonas* strains against the root-knot nematode *M. incognita* (4000 J2/pot) using cucumber seedlings grown in test soil for four weeks (n = 8). Root gall index was scored according to Zeck’s [31] 0–10 scale, where 0 indicates no galls and 10 indicates dead roots. Error bars represent standard deviations of replicates. Significant differences are indicated by an asterisk, calculated by one-way ANOVA with post-hoc Tukey HSD test.

Only cucumber roots grown for four weeks in nematode-infested soil and treated with the 105 or 108 strains showed a significant reduction in root galling compared to cucumbers grown in untreated soil or with other *Pseudomonas* strains (Figure 5). The results showed no significant effect on root gall formation for bacterial strains 102, 112, 119, and 157. It may be that the application method used reduced the RKN control performance of the strains tested, as suggested in previous publications [58].

**Figure 6:**
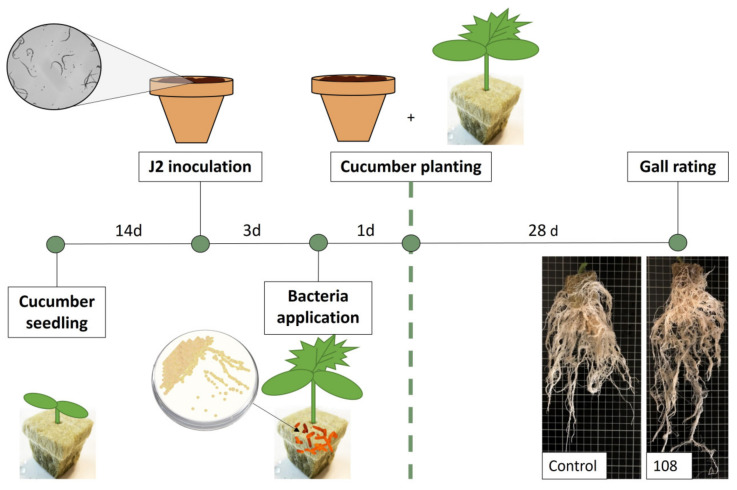
Selected *Pseudomonas* strains were tested as root protectants against *M. incognita* J2 by applying bacterial suspensions to cucumber seedlings grown in mineral wool one day before planting in nematode-infested soil (4000 J2/pot). Cucumber plants were grown for 28 days and root galls were rated according to Zeck’s [31] 0–10 scale, where 0 indicates no galls and 10 indicates dead roots.

The selected *Pseudomonas* strains 102, 105, 108, 119, and 157 were further tested as a root bale treatment. Cucumber seedlings grown on mineral wool plugs (Figure 6) were planted in *M. incognita*-infested soil. Prior to planting, the mineral wool was inoculated with the selected strains.

**Figure 7:**
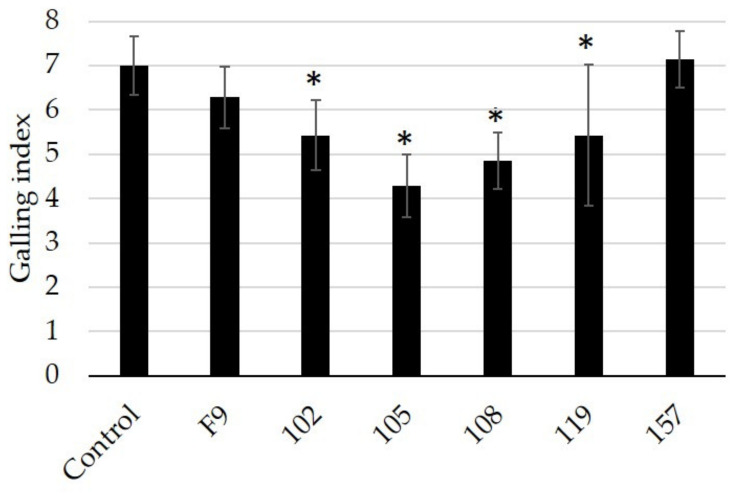
Testing of selected *Pseudomonas* strains as protectants of cucumber roots (grown in mineral wool) against the root-knot nematode *M. incognita* (n = 8). Root galls were scored according to Zeck’s [31] 0–10 scale, where 0 indicates no galls and 10 indicates dead roots. Error bars represent standard deviations of replicates. Significant differences are indicated by an asterisk according to a one-way ANOVA with post-hoc Tukey HSD test.

*Pseudomonas* strains 102, 105, 108, and 119 showed a significant reduction in root galling compared to control plants (Figure 7). As in the previous experiments, *Pseudomonas* strains 105 and 108 showed the strongest reduction in the root galling index. *Pseudomonas* spp. are known to colonize plant roots [26,57]; therefore, the bacterial treatment of the mineral wool may have promoted the bacterial colonization of the cucumber roots, resulting in protection against *M. incognita* infection.

The ability to control *M. incognita* J2 using tomato as an indicator plant was tested with the most promising isolates, *Pseudomonas* strains 105 and 108, and the negative control, *P. orientalis* F9. Two suspensions with an OD_600_ of 0.5 or 1.5 were compared (Table 1).

Root gall formation in tomatoes treated with strain 105 or 108 was reduced regardless of the cell concentration, but a significant reduction in root galling was only achieved with the OD_600_ = 1.5 bacterial suspension.

Previous studies have shown that *Pseudomonas fluorescens* strain CHA0 responds specifically to plant species, age, and genotype when tested against *M. incognita* [59]. Therefore, we hypothesized that *Pseudomonas* strains 105 and 108 may share a similar trait as CHA0 and act somewhat differently on different crop plants.

**Figure 8:**
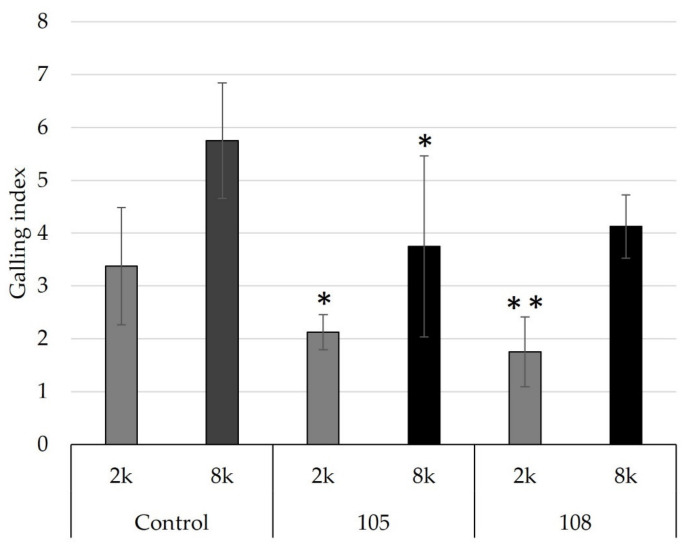
Control efficacy of *Pseudomonas* strains 105 and 108 at low or high *M. incognita* population densities. Low (2k: 2000 J2/pot) or high (8k: 8000 J2/pot) RKN densities were tested, using tomato plants as indicators. The root gall index was determined according to Zeck’s [31] 0–10 scale, where 0 indicates no galls and 10 indicates dead roots. Error bars represent standard deviations of replicates. Significant differences are indicated by an asterisk according to a one-way ANOVA with post-hoc Tukey HSD test (** *p* < 0.01, * *p* < 0.05).

To evaluate whether *Pseudomonas* strains 105 and 108 maintained their control effect at different nematode population densities, their effect was tested in pots inoculated with 2000 or 8000 J2/pot. Both strains showed control of RKN in soil. However, in soil with a higher nematode population density (8000 J2), only strain 105 significantly reduced root gall formation in tomato (Figure 8). Despite the fact that RKN control was significant in our experiments, the control effect was not as strong as, for example, that obtained for *Pseudomonas simiae* strain MB751. This strain promoted up to an 80% reduction in root galling from 6.3 to 1.2 [60]. However, when compared to the recent study by Zhao et al. [57], the control effect of strains 105 and 108 was stronger than that observed for *Pseudomonas protegens* strains Sneb1997 or Sneb2001.

The beneficial effect of RKN control by *Pseudomonas* 105 and 108 strains was also observed on tomato plant height development under soils infested with low (2000 J2/pot) and high (8000 J2/pot) population densities of *M. incognita* (Supplementary Figure S1). The tomato plant height was lower for plants grown in *M. incognita*-infested soil than for plants grown in *M. incognita*-infested soil but treated with *Pseudomonas* 105 or 108 strains. However, plants performed best when the soil was not inoculated with *M. incognita*. Similar results were observed when the biological nematicide BioAct or the chemical nematicide fluopyram were applied, as neither nematicide was able to restore the same yield and plant growth as *M. incognita*-free soil [61]. Based on our experimental design, we cannot conclude that *Pseudomonas* strains 105 or 108 support plant growth and/or seed germination in the absence of nematodes, as reported for *P. protegens* [57].

### 3.3. Sequencing and De Novo Assembly of the Complete Genome of Pseudomonas 105 and 108

To create an optimal basis for the phylogenetic placement of the strains (see below) and for future functional genomics studies to uncover potential mechanisms of action, the complete genomes of both strains were sequenced and de novo assembled. Motivated by recent studies that have demonstrated that Illumina short-read-based genome assemblies can lack important genes that may underlie antagonistic activity, such as non-ribosomal peptide synthetases, phenazine biosynthesis genes, and type six secretion system effectors [62], a combination of long reads (Oxford Nanopore Technologies) and short reads (Illumina) was used (see Methods). Illumina reads were mainly used for polishing and to assemble potential plasmids. The complete genomes of the strains consisted of one chromosome and one plasmid, respectively, and were subsequently annotated with a local installation of the NCBI’s PGAP software (see Methods; Supplementary Table S2). The analysis of the longest repeats indicated that both strains can be classified as difficult to assemble class III genomes [46]; Illumina reads alone would not have allowed us to assemble a complete genome. Finally, an analysis with AntiSmash v6.0.1 [63] predicted several biosynthetic gene clusters of potential relevance (see Supplementary Figure S2A,B), including a lokisin NRP biosynthetic gene cluster, which was shown to exert anti-fungal activity [64]. Moreover, a lokisin derivative was produced in a multispecies bacterial community where the gene and metabolite expression changed depending on the composition [65], underlining the relevance of multispecies consortia.

### 3.4. Phylogenetic Analysis of Pseudomonas Strains 105 and 108

**Figure 9:**
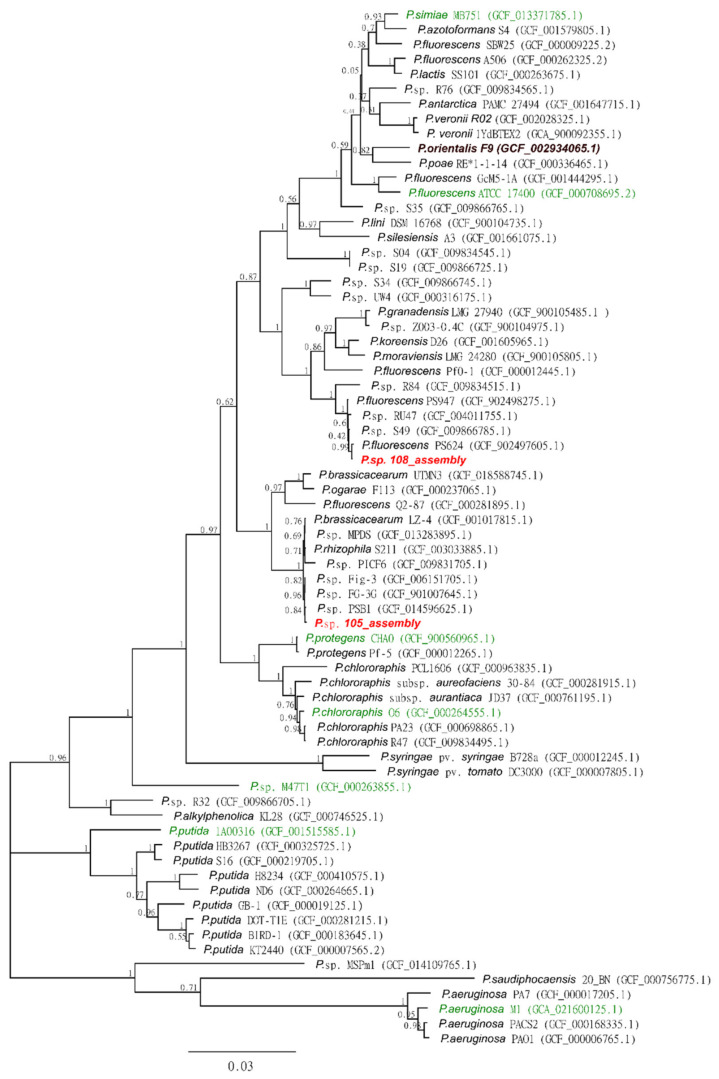
Phylogenetic tree based on 16 core genes of *Pseudomonas* strains for the taxonomic placement of the studied *Pseudomonas* strains 105 and 108 (marked in red) inferred by the Maximum Likelihood method (see Methods). The bootstrap values shown for each node were obtained from 100 bootstrap runs. The genomes sequenced for *Pseudomonas* strains previously reported to contain nematicidal properties are marked in green (Supplementary Table S3). *P. orientalis* F9, used as a negative control for the experiments, is marked in brown. The scale of the branch length units at the bottom of the tree indicates the number of nucleotide substitutions per site.

Phylogenetic analysis using 68 *Pseudomonas* reference strains available in the NCBI database confirmed that both bacterial isolates, 105 and 108, belonged to the genus *Pseudomonas*. Both isolates clustered within the *P. fluorescence* subgroup together with previously identified *Pseudomonas* isolates with potential for nematode biocontrol (Figure 9, Supplementary Table S3). However, strains 105 and 108 were placed in separate subclades of the *P. fluorescence* group.

Strain 105 was closely grouped with the characterized *Pseudomonas brassicacearum* (GCA_001017815) and *Pseudomonas rhizophila* (GCA_003033885), while strain 108 was grouped with characterized *P. fluorescence* strains (Figure 9). However, with bootstrap support of 0.63, the branching of both clades was considered low. The majority of the knots in the tree were between 0.95 and 1, indicating that the phylogenetic tree was robust.

Interestingly, this phylogenetic analysis showed that the *Pseudomonas* isolates used in the present study clustered together with *Pseudomonas* strains previously tested for potential RKN control (Figure 9, Supplementary Table S3 [56,59,66,67,68,69,70,71,72]). For example, *P. simiae* MB751, *P. fluorescens* ATCC-17400, *P. protegens* CHA0, *Pseudomonas putida* 1A00316, and *Pseudomonas aeruginosa* showed nematicidal properties against the RKN *M. incognita* and/or *Meloidogyne javanica* [56,59,60,66,67,68,69,70]. Only *Pseudomonas* chlororaphis O6 has been successfully tested against a temperate RKN, *Meloidogyne hapla* [71].

The mode of action has only been investigated in a few studies. For example, *P. simiae* MB751 produces a cyclic dipeptide with nematicidal properties, which is noteworthy as some fungi have also been reported to produce macrocyclic peptides with nematicidal properties against *M. incognita* [73]. *P. putida* 1A00316, isolated from Antarctic soil, has been shown to produce volatile nematicides [56]. However, based on phylogenetic analysis alone, no conclusions can be drawn about the mode of action of the isolates tested in this study.

Based on our experiment, the selected *Pseudomonas* strains were able to protect cucumber root bales (Figure 8) more efficiently than when applied to soil with a higher OD_600_ of 1.5 (Table 1). Therefore, we hypothesize that the protective properties of *Pseudomonas* strains 105 and 108 are related to root colonization.

Overall, the phylogenetic analysis shows that the *Pseudomonas* strains used in this study cluster not only among the RKN control strains but also among other biocontrol strains, such as the *P. fluorescens* strain 8GCA_902497605, a potato-pathogen-inhibiting strain, or other *Pseudomonas* strains isolated from the rhizosphere and phyllosphere of potato plants, R84 and S49, which have been reported to inhibit the growth of *Phytophthora infestans* mycelia [51].

Therefore, it may be worthwhile to test *Pseudomonas* strains 105 and 108 as biocontrol agents against other important plant-pathogenic nematodes and agricultural fungal and oomycete pathogens. The release of the complete de-novo-assembled genome sequence will serve as an important basis to identify the mechanism(s) of action against various plant pathogens in future studies. On a broader scale, the assay system developed here should also allow the testing of mixtures of different strains with biocontrol activity for robust and potentially even synergistic effects.

## 4. Conclusions

This study serves as a proof-of-principle for the identification of potentially promising biocontrol strains against RKN, with the help of this newly developed screening method.

The straightforward and reliable combination of screening assays resulted in the isolation of *Pseudomonas* strains that controlled *M. incognita* in vitro and in soil.

Further investigation of *Pseudomonas* strains 105 and 108 demonstrated their potential to control *M. incognita* under greenhouse conditions with varying RKN population densities. The application of the *Pseudomonas* strains is versatile, as demonstrated by their efficacy when being used as either soil or root treatments.

Phylogenetic analyses revealed that both *Pseudomonas* strains clustered in the *Pseudomonas fluorescens* group alongside previously described plant pathogens and RKN antagonists, but apart from each other.

Future investigations of the selected *Pseudomonas* candidates for RKN control will need to demonstrate their antagonistic potential in larger greenhouse trials and/or during field applications, investigating the influence of not only biotic but also abiotic factors on the maintenance of their biocontrol potential. Elucidation of the mechanisms of action is now possible given the availability of complete genome sequences.

## Figures and Tables

**Table 1:** Evaluation of tomato root gall rating 4 weeks after transplanting into soil containing 4000 *M. incognita* J2/pot and treated with *Pseudomonas* strain F9, 105, or 108 at OD_600_ of 0.5 or 1.5.

Treatment	Tomato Root Gall Index	
	OD_600_ 0.5	OD_600_ 1.5
Control	4.55 ± 0.52	4.82 ± 0.57
F9	4.6 ± 1.12	5.00 ± 0.53
105	4.05 ± 1.1	3.86 ± 0.64 *
108	4.12 ± 1.2	3.29 ± 1.16 *

* Significant differences compared to control plants (non-treated nematode-infested soil).

## Data Availability

Data are contained within the article and appendices. The complete genome sequences of the two *Pseudomonas* isolates are available from the NCBI under bioproject PRJNA975707 (P105) and PRJNA975710 (P108) and with the accession numbers CP126693-CP126694 (P105) and CP126691-CP126692 (P108).

## Supplementary Materials

The following supporting information can be downloaded at: https://www.mdpi.com/article/10.3390/microorganisms11082011/s1, Figure S1: Effect of bacterial strains on the height of tomato plants grown in nematode-infected soil; Table S1: Selected bacterial strains used in a screen to identify antagonistic activity against *Meloidogyne incognita*; Table S2: Selected genome features of *Pseudomonas* spp. 105 and 108; Figure S2: Graphical output of the AntiSmash prediction server (v.6.0.1) for the two *Pseudomonas* isolates; Table S3: List of genome sequences of available for *Pseudomonas* strains that have been linked to plant-parasitic nematode control.
