# Supplementary material for: A Novel Robust Screening Assay Identifies Pseudomonas Strains as Reliable Antagonists of the Root-Knot Nematode Meloidogyne incognita
Source: Microorganisms. 2023 Aug 4;11(8):2011. doi: 10.3390/microorganisms11082011 (PMC10459205; doi:10.3390/microorganisms11082011)
Supplement: Supplementary file 1 [file microorganisms-11-02011-s001.zip › microorganisms-2517266-supplementary.pdf]

Supplementary Data

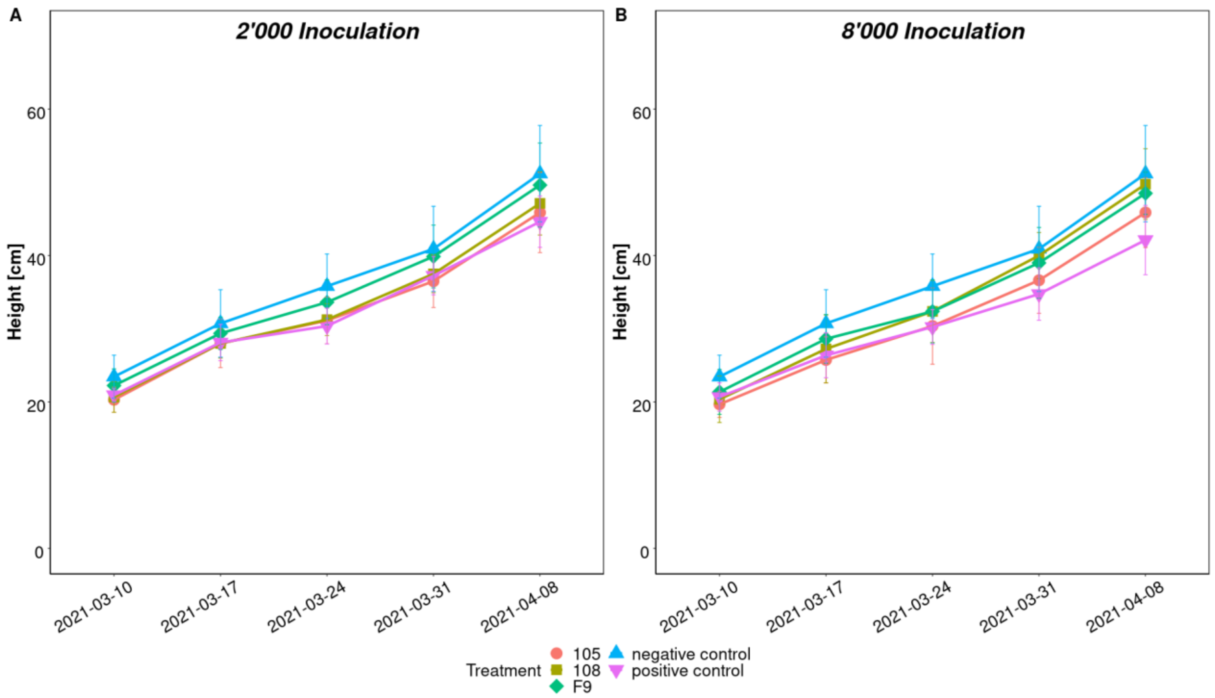

Supplementary Figure S1. Effect of bacterial strains on the height of tomato plants grown in nematode infected soil. Comparison of tomato plant height after growing on nematode-free soil (negative control), and soil inoculated with 2000 J2/pot (A) or 8000 J2/pot (B). Positive control: only J2 infected soil; infected soil treated with *Pseudomonas* strain 105 (105), strain 108 (108), or *P. orientalis* F9 (negative control for bacterial antagonism).

A)

chromosome_1 (Pseudomonas)

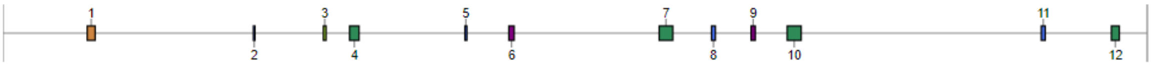

Region	Type	From	To	Most similar known cluster		Similarity
Region 1.1	arylpolyene	462,852	506,463	APE Vf	Other	45%
Region 1.2	RiPP-like	1,375,648	1,384,426			
Region 1.3	NAGGN	1,760,159	1,775,035			
Region 1.4	NRPS	1,904,133	1,955,986	pyoverdin	NRP	10%
Region 1.5	RiPP-like	2,537,606	2,548,493			
Region 1.6	betalactone	2,778,975	2,808,222	fengycin	NRP	20%
Region 1.7	NRPS	3,604,717	3,679,897	herboxidiene	Polyketide	2%
Region 1.8	ranthipeptide	3,892,309	3,913,739	pyoverdin	NRP	8%
Region 1.9	betalactone	4,109,646	4,132,871	fengycin	NRP	13%
Region 1.10	NRPS	4,308,070	4,384,915	pyoverdin	NRP	19%
Region 1.11	redox-cofactor	5,703,688	5,725,853	lankacidin C	NRP + Polyketide	13%
Region 1.12	NRPS-like	6,089,092	6,132,511	fragin	NRP	37%

No secondary metabolite regions were found in these records:
plasmid_1 (Pseudomonas)

B)

chromosome_1 (Pseudomonas)

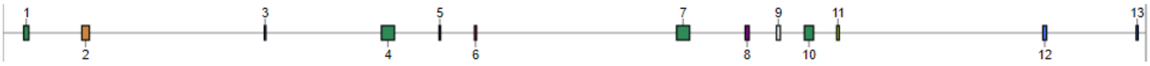

Region	Type	From	To	Most similar known cluster		Similarity
Region 1.1	NRPS-like	120,935	150,668	fragin	NRP	37%
Region 1.2	arylpolyene	458,295	501,899	APE Vf	Other	40%
Region 1.3	RiPP-like	1,517,281	1,526,102			
Region 1.4	NRPS	2,196,293	2,271,884	pyoverdin	NRP	19%
Region 1.5	RiPP-like	2,530,539	2,539,657			
Region 1.6	siderophore	2,736,213	2,748,063			
Region 1.7	NRPS	3,908,819	3,984,107	lokisin	NRP	92%
Region 1.8	betalactone	4,306,598	4,329,863	fengycin	NRP	13%
Region 1.9	blactam	4,488,447	4,510,312	tabtoxin	Other	27%
Region 1.10	NRPS	4,650,128	4,703,126	pyoverdin	NRP	17%
Region 1.11	NAGGN	4,837,515	4,852,368			
Region 1.12	redox-cofactor	6,033,380	6,055,536	lankacidin C	NRP + Polyketide	13%
Region 1.13	RiPP-like	6,575,007	6,585,852			

No secondary metabolite regions were found in these records:
plasmid_1 (Pseudomonas)

Supplementary Figure S2. Graphical output of the AntiSmash prediction server (v.6.0.1) for the two sequenced *Pseudomonas* isolates. Regions with potential biosynthetic gene clusters are shown along with the type, genomic region and most similar known cluster. A) *Pseudomonas* 105 genome assembly. B) *Pseudomonas* 108 genome assembly.

Supplementary Table S1. Selected bacterial strains used in a screen to identify antagonistic activity against *Meloidogyne incognita*. The identification of the strains is based on MALDI-TOF analysis [30] and 16S rRNA sequencing.

Bacterial strain	*Species*	*Source*	*Origin/Reference*
F9	*Pseudomonas orientalis*	Apple flower	Zengerer et al., 2019
10	*Arthrobacter* spp.	Red beet roots	This study, Switzerland
18	*Bacillus* spp.	Red beet roots	This study, Switzerland
20	*Arthrobacter* spp.	Red beet roots	This study, Switzerland
65	*Bacillus* spp.	Red beet roots	This study, Switzerland
68	*Bacillus* spp.	Red beet roots	This study, Switzerland
78	*Bacillus* spp.	Red beet roots	This study, Switzerland
93	*Bacillus* spp.	Red beet roots	This study, Switzerland
101	*Pseudomonas* spp.	Red beet roots	This study, Switzerland
102	*Pseudomonas* spp.	Red beet roots	This study, Switzerland
103	*Pseudomonas* spp.	Red beet roots	This study, Switzerland
104	*Pseudomonas* spp.	Red beet roots	This study, Switzerland
105	*Pseudomonas* spp.	Red beet roots	This study, Switzerland
106	*Pseudomonas* spp.	Red beet roots	This study, Switzerland
108	*Pseudomonas* spp.	Red beet roots	This study, Switzerland
110	*Pseudomonas* spp.	Red beet roots	This study, Switzerland
111	*Pseudomonas* spp.	Red beet roots	This study, Switzerland
112	*Pseudomonas* spp.	Red beet roots	This study, Switzerland
113	*Pseudomonas* spp.	Red beet roots	This study, Switzerland
114	*Pseudomonas* spp.	Red beet roots	This study, Switzerland
115	*Pseudomonas* spp.	Red beet roots	This study, Switzerland
116	*Pseudomonas* spp.	Red beet roots	This study, Switzerland
118	*Pseudomonas* spp.	Red beet roots	This study, Switzerland
119	*Pseudomonas* spp.	Red beet roots	This study, Switzerland
121	*Pseudomonas* spp.	Red beet roots	This study, Switzerland
122	*Pseudomonas* spp.	Red beet roots	This study, Switzerland
123	*Pseudomonas* spp.	Red beet roots	This study, Switzerland
126	*Pseudomonas* spp.	Red beet roots	This study, Switzerland
127	*Pseudomonas* spp.	Red beet roots	This study, Switzerland
128	*Pseudomonas* spp.	Red beet roots	This study, Switzerland
130	*Pseudomonas* spp.	Red beet roots	This study, Switzerland
131	*Pseudomonas* spp.	Red beet roots	This study, Switzerland
132	*Pseudomonas* spp.	Red beet roots	This study, Switzerland
133	*Pseudomonas* spp.	Red beet roots	This study, Switzerland
135	*Pseudomonas* spp.	Red beet roots	This study, Switzerland
137	*Pseudomonas* spp.	Red beet roots	This study, Switzerland
139	*Pseudomonas* spp.	Red beet roots	This study, Switzerland
141	*Pseudomonas* spp.	Red beet roots	This study, Switzerland
142	*Pseudomonas* spp.	Red beet roots	This study, Switzerland
145	*Pseudomonas* spp.	Red beet roots	This study, Switzerland
151	*Pseudomonas* spp.	Red beet roots	This study, Switzerland
154	*Pseudomonas* spp.	Red beet roots	This study, Switzerland
156	*Pseudomonas* spp.	Red beet roots	This study, Switzerland
157	*Pseudomonas* spp.	Red beet roots	This study, Switzerland

Supplementary Table S2. Selected genome features of *Pseudomonas spp*. 105 and 108.
The two *de novo* assembled complete genome sequences were annotated with the NCBI's Prokaryotic Genome Annotation Pipeline (PGAP) [47]. Selected genome features are listed below.

	Pseudomonas spp. 105	*Pseudomonas spp. 108*
No. chromosomes (plasmids)	1 (1)	1 (1)
Genome size (bp)	6'286'012 (chrom.)<br>132'071	6'631'596 (chrom.)<br>300'560
G+C content (%)	60.1% (chrom.)<br>54.7%	59.1% (chrom.)<br>53.8%
Coverage ONT (mean)	182x<br>59x	198x<br>347x
Coverage Illumina	215x (chrom.)<br>71x	192x (chrom.)<br>311x
No. of genes	5756	6207
No. of protein-coding genes (CDS)	5670	6108
No. of rRNA operons (16S, 23S, 5S)	(6, 5, 5)	(7, 6, 6)
No. of tRNA genes	65	76
No. of ncRNA genes	5	4
No. of pseudogenes	58	38
Length of longest repeat (nucleotides)	8'398	10'198
Genome assembly complexity [72]	class III	class III
Prophages (Phaster prediction score)	Chrom.:<br>1'276'230-1'314'600 (intact: 150)	Chrom.:<br>1'410'097-1'453'871 (intact: 150)

Supplementary Table S3. List of genome sequences of *Pseudomonas* strains that have been linked to plant parasitic nematode control. Selected information is provided.

Assembly accession number	*Pseudomonas* strain	Associated with	Nematode tested	Ref.
GCF_013371785	*P. simiae* MB751	Nematicidal	*M. incognita*	[60]
GCF_000708695	*P. fluorescens* ATCC-17400	Supporting plant performance by phosphate solubilization	*M. javanica*	[66~~7~~]
GCF_900560965 (*GCF_000397205*)	*P. protegens* CHA0	Nematicidal	*M. javanica*, *M. incognita*	[59~~-~~, 67~~8~~-69~~70~~]
GCF_000264555	*P. chlororaphis* O6	Nematicidal	*M. hapla*	[71~~3~~]
GCA_000263855	*Pseudomonas sp*. M47T1	Nematicidal	*B. xylophilus*	[72~~4~~]
GCF_001515585	*P. putida* 1A00316	Nematicidal	*M. incognita*	[56]
GCA_021600125	*P. aeruginosa*	Nematicidal	*M incognita*	[70~~1~~]
